# Case report: A rare case of bilateral Leber’s idiopathic stellate neuroretinitis

**DOI:** 10.3389/fmed.2024.1364751

**Published:** 2024-03-19

**Authors:** Wei He, Panli Tang, Hongbin Lv, Lifeng Qiao

**Affiliations:** ^1^Department of Ophthalmology, Sichuan Taikang Hospital, Chengdu, China; ^2^Department of Cardiothoracic Surgery, Sichuan Taikang Hospital, Chengdu, China; ^3^Department of Ophthalmology, Affiliated Hospital of Southwest Medical University, Luzhou, China; ^4^Department of Ophthalmology, Sichuan Academy of Medical Science & Sichuan Provincial People's Hospital, School of Clinical Medicine, University of Electronic Science and Technology of China, Chengdu, China

**Keywords:** neuroretinitis, glucocorticoid, fundus fluorescein angiography, Leber’s idiopathic stellate neuroretinitis, diagnosis

## Abstract

**Background:**

Leber’s idiopathic stellate neuroretinitis (LISN) is a rare disease characterized by disk edema, peripapillary and macular hard exudates, and often, the presence of vitreous cells. To enhance clinical understanding of the disease, a retrospective analysis was conducted on a patient diagnosed with LISN at our hospital, and discussions were held regarding its diagnosis and treatment.

**Methods:**

We reviewed the medical records of a 26-year-old male patient whose main complaint was a decrease in visual acuity of both eyes for 4 days, which had worsened over the last day. After systemic examination, fundus fluorescein angiography, and indocyanine green angiography, the patient was diagnosed with LISN in both eyes. After treatment with glucocorticoids, the patient’s vision showed a significant improvement.

**Results:**

Upon admission, the visual acuity of both eyes was: VOD 0.05, VOS 0.25. After 5 days of treatment, the visual acuity of both eyes was: VOD 0.25, VOS 0.4. After 1 month of follow-up, the visual acuity of both eyes was: VOD 0.4, VOS 0.6. After 5 months of follow-up, the patient’s vision improved to VOD 0.6, VOS 0.8.

**Conclusion:**

The cause of LISN remains unidentified. It is essential to rule out diseases exhibiting similar clinical signs but possessing a clear etiology. The primary treatment approach involves glucocorticoid-based anti-inflammatory therapy, potentially supplemented with antibiotics, antivirals, vasodilators, and traditional Chinese medicine. This disease is usually self-limiting and generally carries a favorable prognosis.

## Introduction

LISN is a condition characterized by swelling of the optic nerve and adjacent nerve fibers, resulting in stellate hard exudates in the macular area (1). While it typically manifests in the 30–40 age bracket, it also occurs with similar frequency in children. The cause of this disease is unknown, but infectious causes, such as syphilis, Lyme disease, cat-scratch disease, toxoplasmosis, and viral infection, should be considered. Instances of retinopathy featuring star-shaped formations due to known causes are prevalent, yet literature contains limited descriptions of idiopathic Leber’s stellate retinopathy cases. Hence, we present a case study involving a patient in whom the disease diagnosis has been established.

## Case description

**Figure 1:**
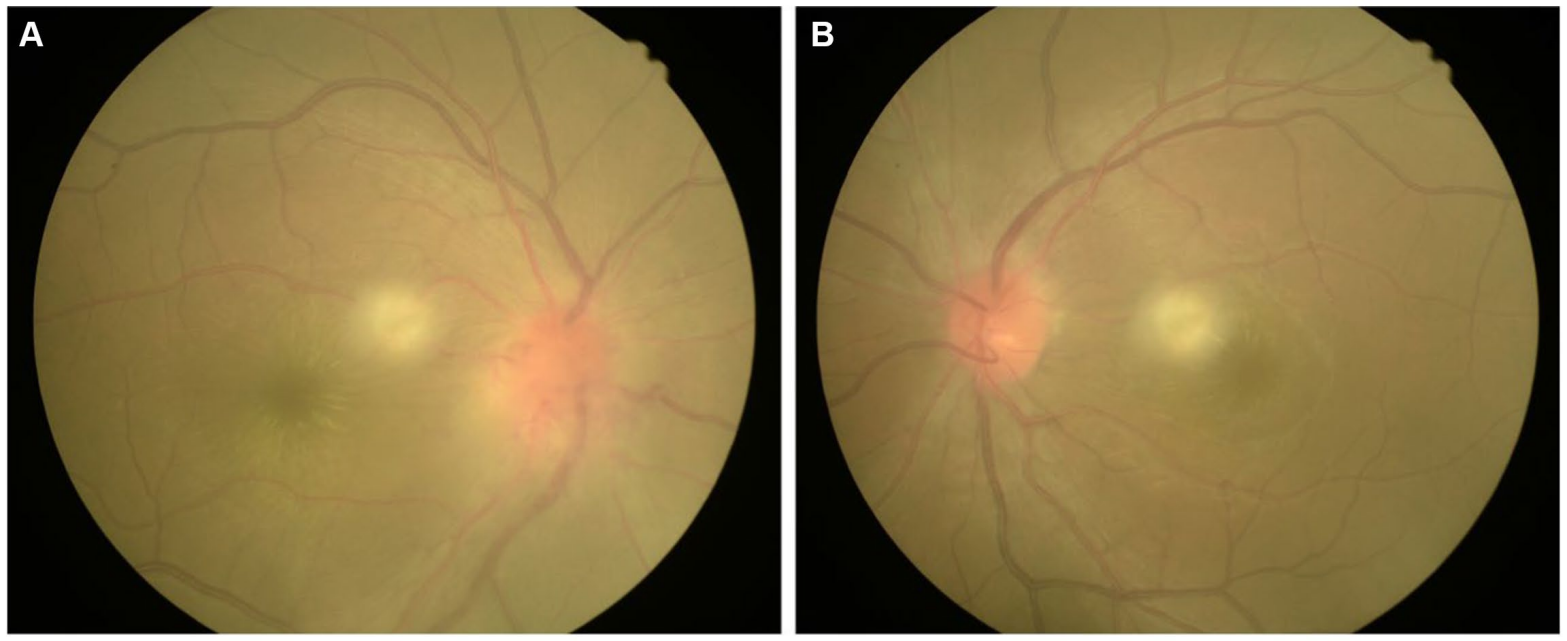
Color fundus images of the eyes of the patient with Leber’s idiopathic stellate neuroretinitis. **(A)** The right eye; **(B)** The left eye.

A 26-year-old man consulted for a decline in visual acuity. He had a history of blurred vision in both eyes for 4 days without pain or redness. The patient reported having a “cold” 5 days before admission. 4 days prior to admission, sudden vision loss in both eyes occurred accompanied by distorted vision, eye movement pain, and distention of the eyes, persisting without improvement. The patient’s medical history revealed no hypertension, diabetes, hepatitis, tuberculosis, surgeries, traumas, blood transfusions, or cat-scratch disease, nor any history of food or drug allergies. Upon admission, ophthalmic examination revealed: VOD 0.05, VOS 0.25. Bilateral conjunctiva showed no congestion or swelling, sclera lacked jaundice, cornea was transparent, anterior chamber depth was normal, pupils were round and centered, approximately 3 mm in diameter, reactive to light. The lenses were clear, and fundus examination showed a red optic disk with blurred margins and “stellate” hard exudates in the macular area (Figure 1). Intraocular pressures were measured as 16 mmHg in the right eye and 15 mmHg in the left eye.

**Figure 2:**
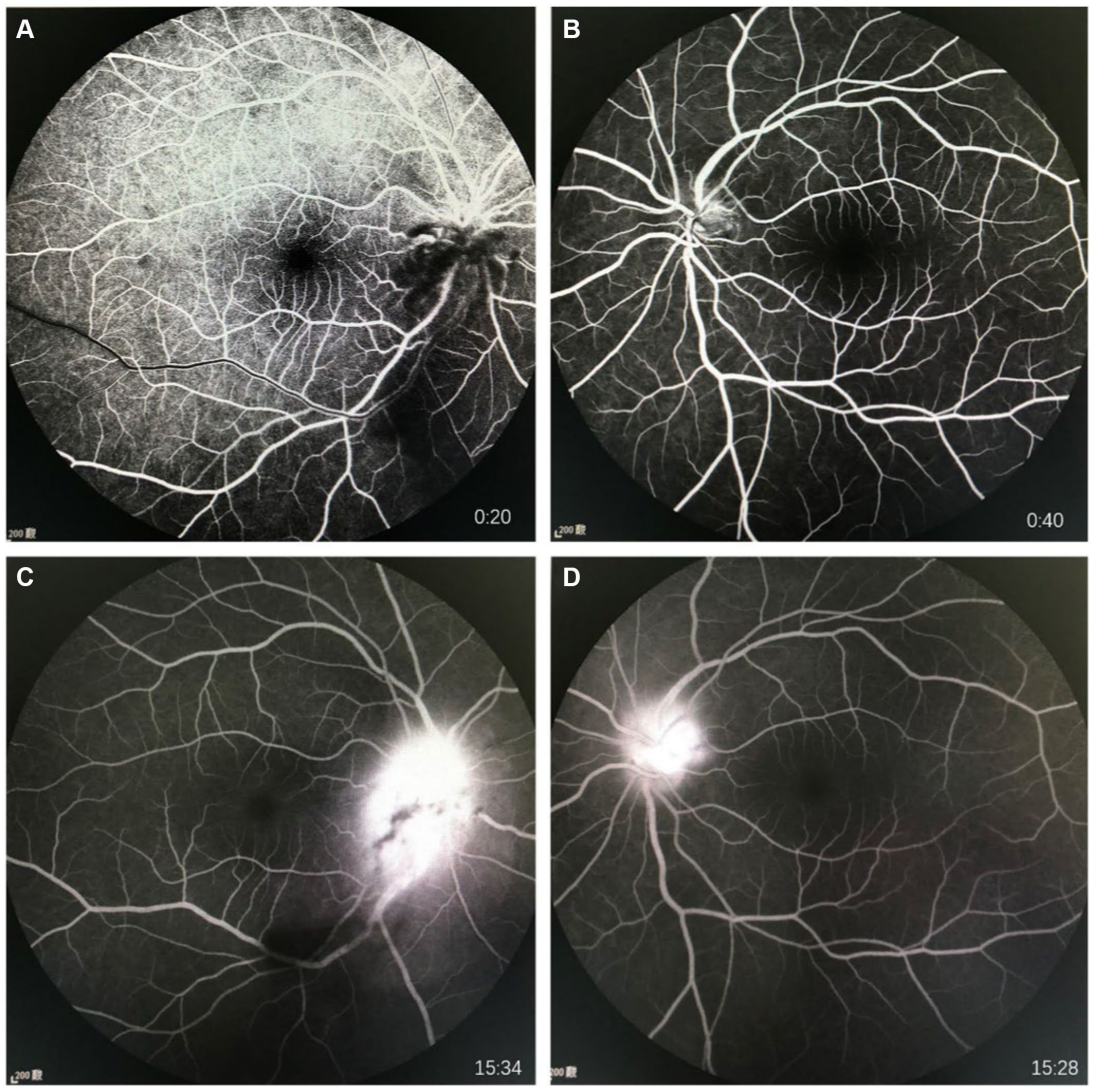
FFA images of the eyes of the patient. **(A)** Shows early stage of FFA of right eye; **(B)** Shows early stage of FFA of left eye; **(C)** Shows late stage of FFA of right eye; **(D)** Shows late stage of FFA of left eye.

The general clinical examination showed no notable findings. Laboratory tests indicated no signs of inflammatory syndrome, with normal levels observed for blood count, electrolytes, C-reactive protein, and erythrocyte sedimentation rate. Peripheral blood cultures for bacteria, influenza nine-plex test and test result for COVID-19 were negative. Urine analysis, liver and kidney function tests, and coagulation function showed no significant abnormalities. Serological tests for cat scratch disease (ELISA for *Bartonella henselae*), syphilis (VDRL and fluorescent treponemal antibody-absorption tests), and toxoplasmosis (serologic testing for anti-Toxoplasma IgG and IgM) all returned negative results. Similarly, tests for tuberculosis, including chest radiography and purified protein derivative (PPD) test, were negative. Magnetic resonance imaging (MRI) of the brain and orbits was performed, revealing no abnormalities. The results of VEP showed that in the right eye, the P100 wave latency was prolonged with low amplitude, while no P100 wave was recorded in the left eye. Fluorescein angiography (FFA) showed retinal circulation time around 15 s, with venous laminar flow seen at 20 s. The inferior large vascular arch showed slightly delayed circulation compared to the upper part. In the late phase of the contrast agent, both optic disks exhibited intense fluorescence, notable accumulation of fluorescein leakage. No abnormal fluorescence was observed in the macular area or peripheral retina (Figure 2).

**Figure 3:**
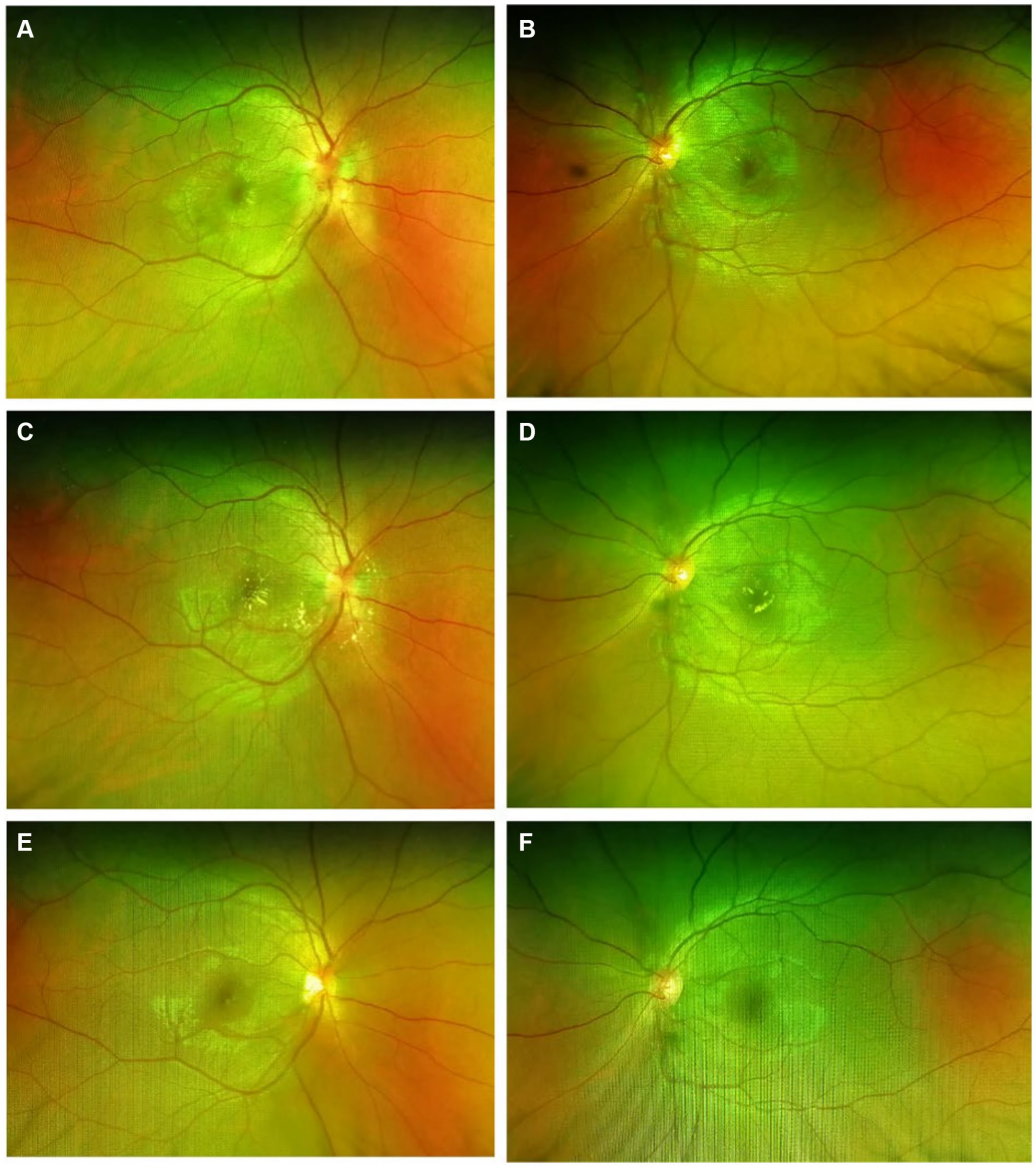
Scanning laser fundus photography of the eyes showed gradual reduction in optic disk congestion and edema, with a gradual complete absorption of stellate exudates in the macular area. **(A,B)** Immediate post-hospital; **(C,D)** 1 month after discharge; **(E,F)** 5 months after discharge.

**Figure 4:**
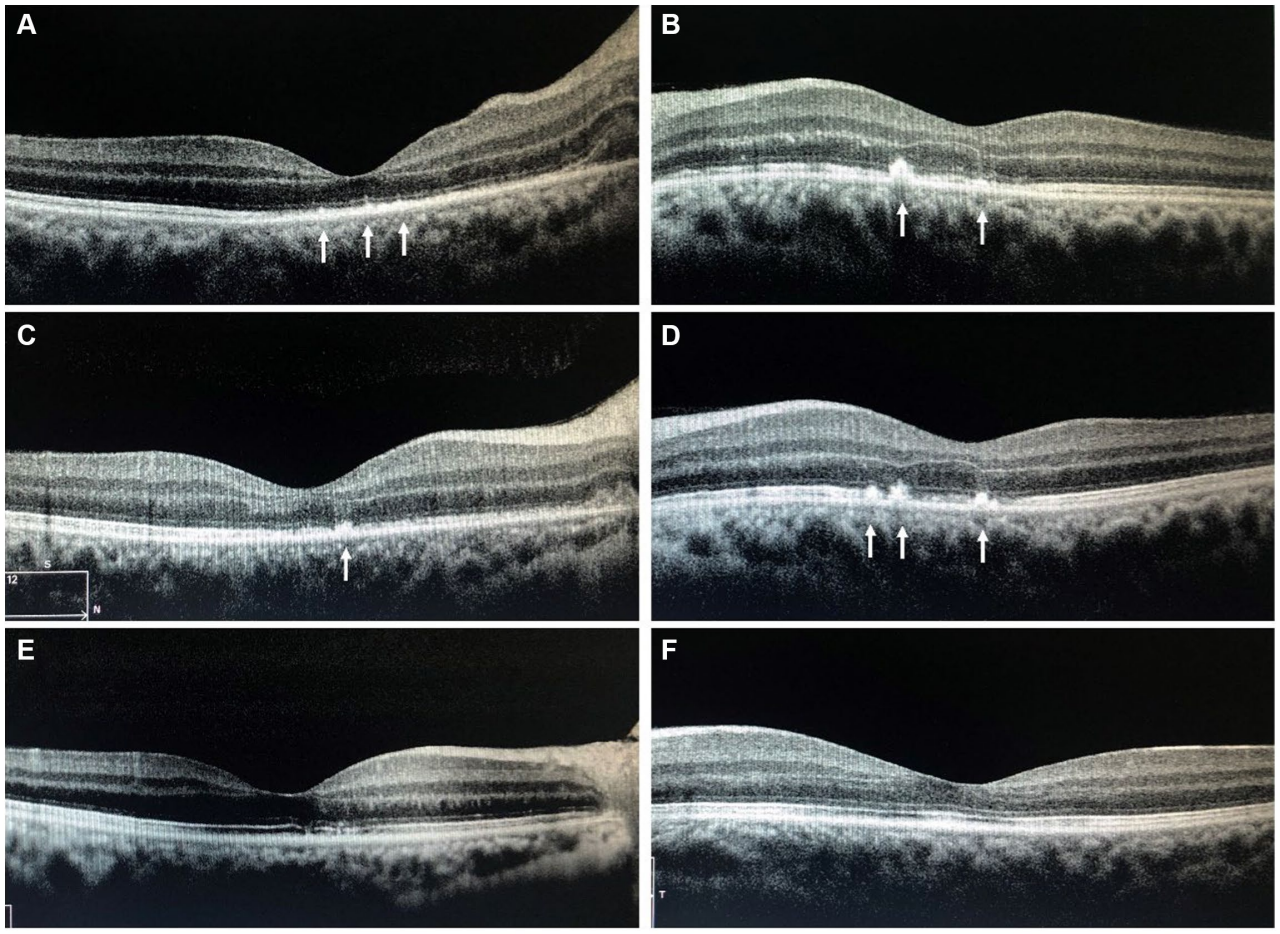
The OCT of the macula shows the gradual complete absorption of the punctate hyper-reflective signals indicated by the arrows. **(A,B)** Immediate post-hospital; **(C,D)** 1 month after discharge; **(E,F)** 5 months after discharge; OCT, Optical Coherence Tomography.

Considering the patient’s medical history, clinical signs, and auxiliary examinations, the diagnosis is bilateral LISN. Treatment involved intravenous infusion of methylprednisolone sodium succinate/methylprednisolone 1 g for 5 days, oral methylcobalamin 500 μg three times a day for 5 days, and oral sucralfate suspension 10 mL three times a day for 5 days. Post-treatment ophthalmic examination showed right eye VOD 0.25, VOS 0.4. The lenses were clear, with the optic disks showing slight reduction in swelling compared to admission, and “stellate” hard exudates were visible in the macular area. Intraocular pressures were measured as 14 mmHg in the right eye and 16 mmHg in the left eye. Medication upon discharge included oral prednisolone acetate tablets 60 mg, with a weekly taper of 10 mg. 1 month after discharge, during follow-up, the patient reported a significant improvement in vision, with VOD at 0.4 and VOS at 0.6. Both optic disks showed reduced congestion and swelling, and partial absorption of stellate exudates in the macular area. Maintenance treatment continued with oral prednisolone acetate tablets at 20 mg. 5 months later during re-examination, the patient’s vision had recovered to OD 0.6 and OS 0.8. Both optic disk boundaries were clear, without apparent congestion. Complete absorption of stellate exudates in the macular area was observed (Figures 3, 4). Subsequent follow-ups indicated stable vision.

## Discussion and conclusion

Leber’s idiopathic stellate neuroretinitis (LISN) was first proposed by Leber in 1916 (2). He reported 9 cases, all presenting with unilateral vision loss, optic disk edema, and stellate exudates in the macular area. So far, literature reports of LISN have not exceeded 20 cases (3). The cause remains unclear and most cases did not undergo treatment, resulting in good visual recovery and resolution of optic disk edema. In 1984, Dreyer observed similar clinical presentations in patients with optic neuropathy and retinitis, coining the term “LISN” to describe a distinct group of patients characterized by optic disk edema and stellate exudates in the macular area, without other ocular or systemic diseases, and not of viral infectious origin (4). Some researchers suggest that LISN is a virus-induced autoimmune disease affecting the optic nerve head vascular system. Lipid-rich exudates leak from these vessels, spreading from the optic disk to the outer plexiform layer. Initially serous exudates are absorbed, while lipids deposit within the Henle fiber layer, forming stellate exudates (5).

Optic neuroretinitis can occur at any age but is typically more prevalent in children and young adults. It commonly manifests as unilateral vision loss without pain and often lacks other ocular or systemic symptoms. However, the bilateral onset observed in this case is quite rare. The patient’s visual acuity can range from 1.0 to light perception. Fundoscopic examination reveals variable degrees of optic disk edema. Retinal vascular dilation and tortuosity may be present, along with peripapillary retinal edema and exudative retinal detachment. The majority of optic neuroretinitis patients do not have a clear etiology, possibly related to conditions like cat scratch disease, Lyme disease, and toxoplasmosis. It’s also suggested that optic neuroretinitis could be either viral infections or immune-mediated, with research indicating up to 50% of patients having prior viral infections, often involving the respiratory tract and occurring several weeks before the onset of visual symptoms. In this case, the patient had a history of the common cold 5 days before the onset of symptoms, suggesting a potential association with a viral infection.

Typical fundus changes in LISN include optic disk edema and stellate exudates deposited in the Henle layer, forming a characteristic star-shaped or semi-star-shaped appearance. Stellate exudates in the macula typically appear after optic disk edema. In this case, the patient presented with bilateral optic disk congestion, edema, and stellate hard exudates in the macular area, consistent with fundus changes seen in LISN (6).

It’s important to note that LISN is a diagnosis of exclusion, requiring the exclusion of systemic and ocular factors that could cause similar symptoms, such as hypertension, diabetes, cat scratch disease, syphilis, tuberculosis, and more. Based on the patient’s medical history and relevant laboratory tests, all these factors can be ruled out. However, it is worth noting that visual field testing plays a crucial role in diagnosing neuroretinitis. This is because visual field defects may occur in the early stages of neuroretinitis. By quantitatively assessing the extent of visual field damage, doctors can determine the severity of neuroretinitis, thereby formulating more effective treatment plans. However, due to limitations in retrospective studies, we were unable to find visual field examination results in this patient’s medical records. Nevertheless, we still emphasize the importance of this examination in diagnosing neuroretinitis.

Based on the above, the symptoms and signs of this case align with the diagnosis of LISN. Optic neuroretinitis is typically a self-limiting condition. The primary treatment involves anti-inflammatory corticosteroids, sometimes combined with antibiotics, antivirals, vasodilators, and traditional Chinese medicine, such as *Salvia miltiorrhiza* and notoginseng. Due to the unclear etiology, the treatment of this condition mainly focuses on non-specific anti-inflammatory therapy, with glucocorticoids being the preferred medication. Glucocorticoids are considered to alleviate inflammatory responses, particularly during the acute phase of the disease, and are believed to shorten the duration of the illness (7). Stellate exudates in the macular area usually begin to resolve around 1 month after onset, with complete resolution taking around 6–12 months (8). Optic disk edema often resolves completely, though in rare cases, there might be mild residual optic nerve atrophy, with most patients experiencing full visual recovery. In this case, after receiving high-dose corticosteroid therapy, the patient’s vision significantly improved. 1 month after discharge, the optic disk edema noticeably decreased, and 5 months after discharge, the stellate exudates in the macular area had completely resolved.

In conclusion, we present a rare case of LISN in a 26-year-old male patient. The diagnosis of LISN remains challenging due to its idiopathic nature, lacking a definitive etiology. However, thorough investigations excluding known infectious and systemic causes are pivotal in establishing a LISN diagnosis. This case emphasizes the significance of considering LISN as a differential diagnosis in patients presenting with optic disk edema and stellate exudates in the macular area. It underscores the effectiveness of glucocorticoid therapy and the necessity of meticulous exclusion of other potential etiologies, ultimately contributing to improved patient outcomes and visual recovery.

## Data availability statement

The datasets presented in this article are not readily available because of ethical and privacy restrictions. Requests to access the datasets should be directed to the corresponding author/s.

## Ethics statement

Ethical review and approval was not required for the study on human participants in accordance with the local legislation and institutional requirements. Written informed consent from the patients/participants or patients/participants’ legal guardian/next of kin was not required to participate in this study in accordance with the national legislation and the institutional requirements. Written informed consent was obtained from the individual(s) for the publication of any potentially identifiable images or data included in this article.

## Author contributions

WH: Data curation, Investigation, Writing – original draft, Writing – review & editing. PT: Validation, Visualization, Writing – original draft. HL: Conceptualization, Supervision, Validation, Writing – review & editing. LQ: Conceptualization, Investigation, Resources, Supervision, Writing – review & editing.
